# The Transcriptional Aftermath in Two Independently Formed Hybrids of the Opportunistic Pathogen Candida orthopsilosis

**DOI:** 10.1128/mSphere.00282-20

**Published:** 2020-05-06

**Authors:** Hrant Hovhannisyan, Ester Saus, Ewa Ksiezopolska, Toni Gabaldón

**Affiliations:** a Barcelona Supercomputing Centre (BSC-CNS), Barcelona, Spain; b Institute for Research in Biomedicine (IRB), Barcelona, Spain; c Universitat Pompeu Fabra, Barcelona, Spain; d Institució Catalana de Recerca i Estudis Avançats, Barcelona, Spain; University of Georgia

**Keywords:** *Candida orthopsilosis*, hybridization, pathogen, transcriptomics, yeasts

## Abstract

Interspecific hybridization can drive evolutionary adaptation to novel environments. The *Saccharomycotina* clade of budding yeasts includes many hybrid lineages, and hybridization has been proposed as a source for new pathogenic species. Candida orthopsilosis is an emerging opportunistic pathogen for which most clinical isolates are hybrids, each derived from one of at least four independent crosses between the same two parental lineages. To gain insight into the transcriptomic aftermath of hybridization in these pathogens, we analyzed allele-specific gene expression in two independently formed hybrid strains and in a homozygous strain representative of one parental lineage. Our results show that the effect of hybridization on overall gene expression is rather limited, affecting ∼4% of the genes studied. However, we identified a larger effect in terms of imbalanced allelic expression, affecting ∼9.5% of the heterozygous genes in the hybrids. This effect was larger in the hybrid with more extensive loss of heterozygosity, which may indicate a tendency to avoid loss of heterozygosity in these genes. Consistently, the number of shared genes with allele-specific expression in the two independently formed hybrids was higher than random expectation, suggesting selective retention. Some of the imbalanced genes have functions related to pathogenicity, including zinc transport and superoxide dismutase activities. While it remains unclear whether the observed imbalanced genes play a role in virulence, our results suggest that differences in allele-specific expression may add an additional layer of phenotypic plasticity to traits related to virulence in C. orthopsilosis hybrids.

**IMPORTANCE** How new pathogens emerge is an important question that remains largely unanswered. Some emerging yeast pathogens are hybrids originated through the crossing of two different species, but how hybridization contributes to higher virulence is unclear. Here, we show that hybrids selectively retain gene regulation plasticity inherited from the two parents and that this plasticity affects genes involved in virulence.

## Abstract (precis)

How new pathogens emerge is an important question that remains largely unanswered. Some emerging yeast pathogens are hybrids originated through the crossing of two different species, but how hybridization contributes to higher virulence is unclear. Here, we show that hybrids selectively retain gene regulation plasticity inherited from the two parents and that this plasticity affects genes involved in virulence.

## INTRODUCTION

The incidence of human fungal infections has steadily increased during the past decade, leading to recognition of their relevance in global epidemiology (1). Numerous factors may underlie this growing prevalence, including among others, increased number of immunocompromised individuals (elderly people, neonates, HIV patients, etc.) (2), emergence of drug resistance associated with extensive use of antimycotic agents (3, 4), globalization (5, 6), and climate change (7). The rising incidence of mycoses is also coupled to the identification of a larger number of etiological agents, including so-called emergent pathogens that are increasingly identified in clinics (8, 9). In this context, hybridization between different species or lineages has been identified at the origin of several emerging yeast pathogens (5, 10–14).

For some fungal hybrid pathogens, the two parental species have been identified, as in the case of Cryptococcus neoformans × Cryptococcus deneoformans (15), and Cryptococcus neoformans × Cryptococcus gattii (16). For others, one or both (or possibly numerous) parental species are still unknown. For example, for Candida orthopsilosis, only one of the two putative parental lineages has been identified (11, 13), which constitutes a minority (∼7%) of the analyzed clinical strains. In the case of Candida metapsilosis or Candida inconspicua, both parental species remain unknown (10, 12), as all analyzed strains are hybrids. The fact that some parental species of hybrid fungal pathogens are never or rarely identified among clinical isolates suggests that some human pathogens have arisen from nonpathogenic parental organisms (5, 13), or that the parental lineages have been outcompeted by their more-adapted pathogenic hybrid descendants (17–19). In either case, whether interactions between the two distinct subgenomes contribute to emerging properties in the hybrid, such as increased ability to infect humans, is still poorly understood.

At the molecular level, hybridization results in a state often referred as a “genomic shock” (20), in which two diverged genomes which have evolved independently for a certain time are now sharing the same cellular environment. This coexistence can lead to alterations at several levels, including the genome (21), transcriptome (22), or proteome (23), among others (24). Advances in next-generation sequencing have facilitated the study of the genomic aftermath of hybridization, which involves phenomena such as large-scale genome duplications or deletions, homologous recombination, and gene conversion leading to loss of heterozygosity (5, 12, 21, 25, 26).

However, our understanding of the impact of hybridization at the transcriptomic level remains poorly characterized, with few studies performed on industrial or plant saprophyte hybrids (22, 27–30).

A powerful approach to assess the impact of hybridization on the transcriptome is the assessment of allele-specific expression (ASE), a phenomenon in which one of the two alleles of the gene is preferentially expressed over the other one. With current advances in next-generation sequencing technologies, such as transcriptome sequencing (RNA-Seq), ASE can be assessed in a transcriptome-wide manner. Numerous studies of ASE have been performed in recent years, including studies with yeasts (31–33).

To date, no study of the ASE and transcriptomic aftermath of hybridization has been performed in hybrid human pathogens, limiting our insights on how hybridization leads to emergent traits, including virulence. To fill in this gap, here we undertook a transcriptomic analysis of two independently formed hybrid strains from the emerging yeast pathogen Candida orthopsilosis (11, 13). This yeast belongs to the CTG clade and is phylogenetically placed within the C. parapsilosis *sensu lato* species complex alongside with the other opportunistic pathogens C. parapsilosis (34, 35) and C. metapsilosis (36). It has been shown that most (∼93%) of the clinical isolates of C. orthopsilosis are hybrids between parental lineages with ∼5.1% nucleotide divergence. As mentioned above, only one of the two parental lineages has been found among clinical isolates (11). Thus far, the other partner in the hybridization remains unidentified. Notably, these two parental lineages have hybridized several independent times, giving rise to at least four distinct hybrid clades that differ in their levels and patterns of loss of heterozygosis (LOH) (11). Clade 1 comprises strains that underwent extensive LOH, and are thought to derive from a relatively ancient hybridization, while strains in clade 4 are the most heterozygous, with fewer LOH events and are thus assumed to result from a more recent event. Thus, C. orthopsilosis represents an appropriate model to study how hybridization impacts transcription in a natural hybrid pathogen and whether parallel hybridization events result in similar transcriptomic interactions between the two parental subgenomes.

**FIG 1:**
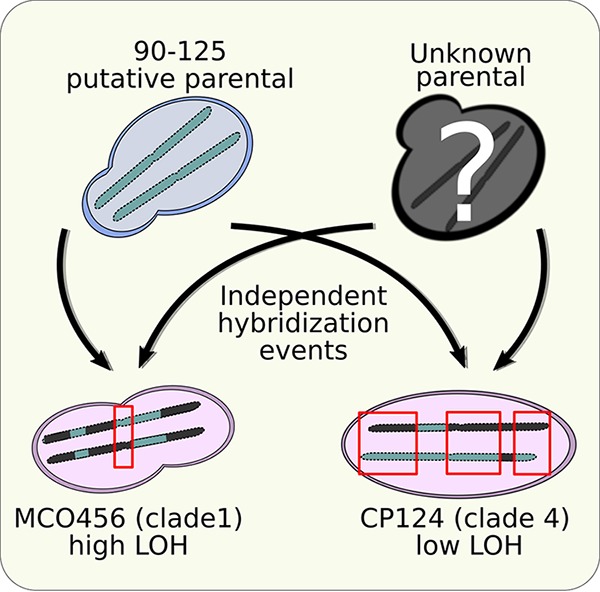
Schematic representation of the experimental design of the study. The C. orthopsilosis 90-125 strain represents a putative parental lineage, which has undergone several independent hybridization events (black arrows) by mating with a second, unknown parental strain. A supposedly more ancient hybridization event has given rise to a hybrid clade, including the MCO456 strain (“high LOH”), which experienced extensive LOH, and a more recent hybridization event has led to the formation of an independent hybrid clade, including CP124 strain (“low LOH”), which contains more heterozygous regions (highlighted with red rectangles).

To shed light on these questions, we conducted ASE analysis using RNA-Seq of two hybrid strains of C. orthopsilosis, each resulting from an independent hybridization event (Fig. 1), that represent the two extremes of LOH extent, namely, MCO456 (clade 1) and CP124 (clade 4). For comparison, we investigated the transcriptome of a highly homozygous strain belonging to one of the putative parental lineages (strain 90-125). To our knowledge, this is the first description of the transcriptomic profiles of parental species of a hybrid yeast that is an opportunistic pathogen of humans.

## RESULTS

To understand the impact of hybridization on gene expression and disentangle the transcriptomic interactions between the two parental subgenomes in the pathogenic hybrid yeast C. orthopsilosis, we performed RNA-Seq of two hybrid strains and a homozygous strain belonging to one of the putative parental lineages (11, 13). Importantly, the two analyzed hybrid strains belong to two independently formed hybrid clades resulting from the mating of the same two parental lineages: strain MCO456 belongs to hybrid clade 1, which underwent extensive LOH, whereas strain CP124 belongs to clade 4, which has limited LOH (Fig. 1). Additionally, we analyzed a strain belonging to one of the putative parental homozygous lineages (strain 90-125) and compared its transcriptomic profile with the hybrids. The summary statistics of our RNA-Seq data, including mapping rates and reproducibility metrics are available in Data Set S1 (tab 1 and tab 2, respectively) in the supplemental material.

10.1128/mSphere.00282-20.3 DATA SET S1 (Tab 1) Summary statistics of RNA-Seq data sets. (Tab 2) Spearman’s correlations for RNA-Seq replicates in the strains studied. (A) CP124, (B) MCO456, (C) 90-125. (Tab 3) List of phased genes in strain MCO456. (Tab 4) List of phased genes in strain CP124. (Tab 5) Read counts for MCO456 strain and 90-125 parental strain. (Tab 6) Read counts for CP124 strain and 90-125 parental strain. (Tab 7) Differential gene expression analysis between the MCO456 hybrid compared to the 90-125 parental strain. Upregulation corresponds to hybrid background. Columns correspond to DESeq2 metrics. (Tab 8) Differential gene expression analysis between the CP124 hybrid compared to the 90-125 parental strain. Upregulation corresponds to hybrid background. Columns correspond to DESeq2 metrics. (Tab 9) Putative functions of differentially expressed genes between MCO456 compared to 90-125 strain. (Tab 10) Putative functions of differentially expressed genes between CP124 compared to 90-125 strain. (Tab 11) Allele-specific expression analysis of MCO456 hybrid. Upregulation corresponds to 90-125 homologs. Columns correspond to DESeq2 metrics. (Tab 12) Allele-specific expression analysis of CP124 hybrid. Upregulation corresponds to 90-125 homologs. Columns correspond to DESeq2 metrics. (Tab 13) Putative functions of allele-specifically expressed genes in MCO456 hybrid. (Tab 14) Putative functions of allele-specifically expressed genes in CP124 hybrid. Download Data Set S1, XLSX file, 0.3 MB. Copyright © 2020 Hovhannisyan et al. 2020 Hovhannisyan et al. This content is distributed under the terms of the Creative Commons Attribution 4.0 International license.

**FIG 2:**
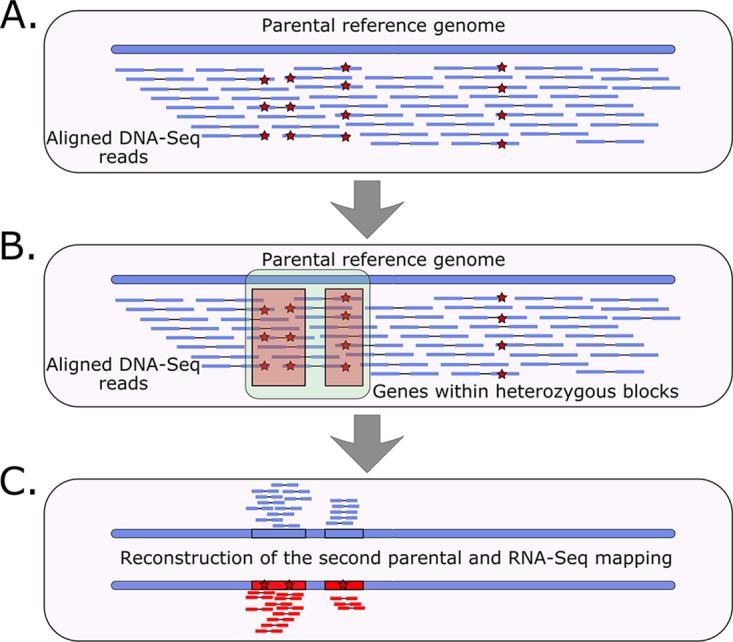
Schematic representation of the bioinformatics approach to assess the allele-specific expression in the hybrid strains. (A) Mapping of DNA-Seq reads to the parental reference genome and further variant calling (red stars represent heterozygous variants). (B) Defining heterozygous blocks (green rectangle) and identifying genes (red rectangles) within the blocks. (C) Inserting the heterozygous variants in the reference genome (second parental reconstruction) and further RNA-Seq read mapping to the partially phased genome.

To perform accurate assignment of RNA-Seq reads to each of the two parental subgenomes in the hybrid, we used the following genome phasing procedure (see Fig. 2 and Materials and Methods for details). Knowing the genome of a relative of one of the putative parents (i.e., strain 90-125), we used it as a reference to map publicly available DNA sequencing (DNA-seq) raw data of the two hybrid strains. We phased (i.e., resolved alternative haplotypes) the heterozygous regions in each of the hybrids by reconstructing the haplotype belonging to the known parental lineage (i.e., having the alleles present in the strain 90-125 sequence) and the alternative haplotype belonging to the unknown parental lineage (i.e., having the heterozygous allele alternative to that in strain 90-125). Using this procedure, we could phase 107 and 590 genes within heterozygous regions for MCO456 and CP124 strains, respectively (Data Set S1, tab 3 and tab 4), from which 71 genes were common between the two strains. As expected, we obtained more phased genes in the clade 4 strain (CP124), which encompasses more heterozygous regions than MCO456 (clade 1). Notably, the overlap of 71 genes in heterozygous regions between the two strains is more than expected by chance as calculated by a hypergeometric test (*P* = 1.011097e−45), which suggests the existence of structural or selective constraints acting on LOH events.

**FIG 3:**
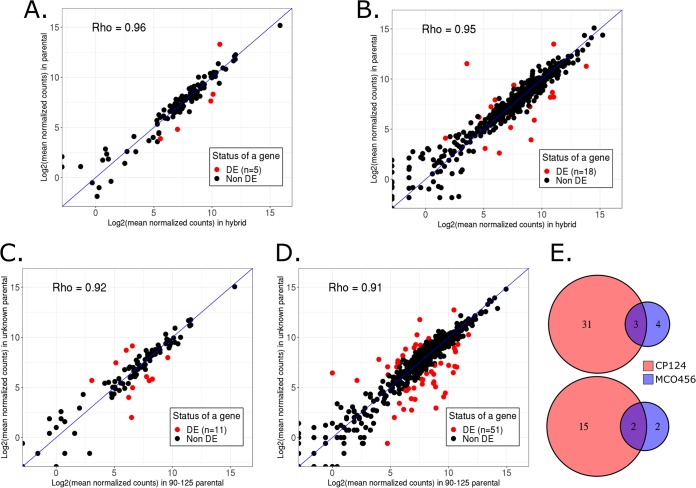
Overall results of differential expression (DE) and allele-specific expression (ASE) comparisons. (A) Correlation between gene expression levels in strains 90-125 and MCO456. (B) Correlation between gene expression levels in strains 90-125 and CP124. (C) ASE analysis for C. orthopsilosis MCO456 strain. (D) ASE analysis for CP124 strain. Scatterplots are based on mean normalized read counts for each gene. DE, differentially expressed. (E) Venn diagrams showing the overlap between ASE genes in both strains: upregulated 90-125 homologs (top) and upregulated homologs of unknown parent (bottom).

Once we obtained the phasing information, we further assessed the levels of allelic expression in hybrids by mapping the RNA-Seq data to the concatenated phased genomes using strict filters for read mapping mismatches (see Materials and Methods). We checked the rates of cross-mapping, i.e., reads that cannot be unambiguously mapped to either parental strain, by mapping the data from the parental strain to the concatenated phased genomes, and observed a negligible proportion of cross-mapping in phased genes (∼0.019% and ∼0.023% for strains MCO456 and CP124, respectively; see Fig. S1 and S2 in the supplemental material). The expression levels in strain 90-125 were obtained by mapping RNA-Seq data directly to the reference genome. Read counts for all strains can be found in Data Set S1, tab 5 and tab 6. Then we performed differential expression (DE) and allele-specific expression (ASE) analysis in both hybrid strains and the parental strain (Fig. 3).

10.1128/mSphere.00282-20.1 FIG S1 Box plots of log_2_ read counts when mapping the RNA-Seq data of C. orthopsilosis 90-125 parental strain to the concatenated reference genome of the MCO456 strain. Numbers above box plots indicate raw read counts. Download FIG S1, TIF file, 0.2 MB. Copyright © 2020 Hovhannisyan et al. 2020 Hovhannisyan et al. This content is distributed under the terms of the Creative Commons Attribution 4.0 International license.

10.1128/mSphere.00282-20.2 FIG S2 Box plots of log_2_ read counts when mapping the RNA-Seq data of C. orthopsilosis 90-125 parental strain to the concatenated reference genome of CP124 strain. Numbers above box plots indicate raw read counts. Download FIG S2, TIF file, 0.2 MB. Copyright © 2020 Hovhannisyan et al. 2020 Hovhannisyan et al. This content is distributed under the terms of the Creative Commons Attribution 4.0 International license.

We first compared the expression levels of strain 90-125 genes with the corresponding genes in heterozygous blocks in the hybrid strains. Such parent-hybrid comparisons (Fig. 3A and B) revealed a limited effect of hybridization on the parental gene expression levels—5 (4.6%) and 18 (3%) DE genes when comparing strain 90-125 with MCO456 and CP124 hybrids, respectively (Data Set S1, tab 7 and tab 8). Most of the differentially expressed genes have unknown roles, with some exceptions (Data Set S1, tab 9 and tab 10). For example, CORT_0B00460 which is upregulated in the hybrid MCO456 background, is predicted to have functions related to metal ion binding and superoxide metabolic processes.

Further, we performed allele-specific gene expression analysis in the hybrids. We detected 11 allele-specifically expressed (ASE) genes in strain MCO456 (10.2% of phased genes; Data Set S1, tab 11), while strain CP124 showed 51 ASE genes (8.6% of phased genes; Data Set S1, tab 12). Putative functions for ASE genes in MCO456 and CP124 strains can be found in Data Set S1, tab 13 and tab 14, respectively. Although the function of most ASE genes was unknown, some have orthologs involved in virulence in other *Candida* pathogens. For both strains, genes related to superoxide dismutase activity (37–39) (CORT_0B00460 in strain MCO456 and CORT_0A12390 in strain CP124) were upregulated for the allele of the unknown parent (37–39). When comparing the parental strain 90-125 with the corresponding homologs in the hybrids, the expression level of CORT_0A12390 was intact upon hybridization, while CORT_0B00460 was upregulated in MCO456.

Moreover, we identified ASE genes related to zinc metabolism, which is one important player in host-pathogen interactions (40–42). The gene CORT_0C02470, potentially involved in zinc transmembrane transport, was upregulated in the hybrid toward the 90-125 parental allele in strain MCO456, while CORT_0E02010, with putative zinc binding activity, is expressed at higher levels from the allele assigned to the unknown parental in strain CP124. For both genes, the expression levels were not altered upon hybridization.

Notably, five genes, CORT_0A04580, CORT_0A09590, CORT_0A03280, CORT_0D04190, and CORT_0E01400, are common ASE genes between the two hybrids, which is higher than expected by chance considering the shared fraction of heterozygous genes (Fig. 3E, *P* = 9.433106e−06). While the functions of the first four genes are unknown, the orthologs of the CORT_0E01400 gene, which expresses the unknown parental allele more, are involved in cellular response to drugs and extracellular region localization (according to *Candida* Genome Database [CGD] annotations).

## DISCUSSION

Here, we investigated the transcriptomic interactions of divergent parental subgenomes in C. orthopsilosis hybrids. To our knowledge, this represents the first such study in a hybrid human opportunistic yeast pathogen. Our experimental design allowed us not only to compare expression of genes in a hybrid genetic background to that in a homozygous parental background but also to assess the extent of convergent evolution in two independently formed hybrid lineages.

In agreement with previous studies of transcriptomic shock in fungal hybrids (22, 30), our results indicate that hybridization has a rather moderate effect on gene expression levels—on average, ∼4% of the genes studied exhibited changes in the expression level upon hybridization compared to the parent. This relatively low level of transcriptomic alteration upon hybridization in yeast hybrids contrasts with the higher levels reported for animals or plants, as noted earlier (30), and reinforcing the idea that yeasts have a comparatively higher capacity than plants and animals to buffer the effects of the transcriptomic shock elicited by hybridization. As more transcriptomic studies on diverse organisms accumulate, how widespread these differences are and what molecular mechanisms may underlie this phenomenon will become clear.

When comparing the results of this study with those of the report assessing transcriptomic shock in an artificial yeast hybrid (30), we noted that the proportion of DE genes (calculated over the total number of genes in heterozygous regions) in natural hybrids is somewhat larger (∼4% in this study) than in the case of the artificial Saccharomyces cerevisiae × Saccharomyces uvarum hybrids (∼1.5%), despite the larger parental divergence and more recent nature of the hybridization. Additionally, we found that the two independently formed C. orthopsilosis hybrids retained a shared subset of genes in heterozygosis, which is larger than expected by chance. Altogether, these results suggest that structural constraints or functional selection may play a role in shaping LOH patterns in these hybrids, with certain genes, including some showing divergent expression patterns, being more likely to be retained in heterozygosis. Moreover, it has been previously reported that LOH events can be driven by selective pressures (25). A plausible scenario is that shared genes in heterozygosis and shared DE genes between the two independently formed hybrids are involved in traits beneficial for the hybrid and are thus maintained through purifying selection. Nevertheless, comparisons from such a limited number of studies must be taken with caution, and we hope that future studies will help to clarify such questions.

When assessing differences in expression between homologous copies in the hybrid (ASE), we found that the fraction of genes with ASE (8.6% in strain CP124 and 10.2% in strain MCO456) was comparatively larger than the fraction of DE genes. In addition, these fractions of ASE genes are slightly higher than that noted for a newly formed S. cerevisiae × S. uvarum hybrid (7.4%), despite the much lower parental divergence in C. orthopsilosis hybrids. Moreover, the fraction of ASE genes is larger in strain MCO456 (10.2% compared to 8.6% in strain CP124) which underwent more extensive LOH. Although more data sets are needed to confirm this trend, this observation suggests that LOH preferentially targets genes that do not display ASE, consistent with the observation above for DE genes. Of note, we did not observe a strong preferential expression of alleles from either one of the parental subgenomes with 34 (∼66%) and 7 (∼63%) of ASE genes expressed higher in the known parental subgenome in CP124 and MCO456 strains, respectively. This is in line with initial comparisons of C. orthopsilosis genomes, which showed no preferential retention of any of the parental genomes in regions undergoing LOH (13). Previous studies in natural (*Epichloë*) and artificial (S. cerevisiae × S. uvarum) fungal hybrids have also reported no strong preferential overexpression of one of the parental subgenomes (22, 29), which suggests this may be a general phenomenon in ascomycete fungal hybrids.

For both C. orthopsilosis hybrid strains, we identified ASE genes involved in processes directly related to virulence, such as zinc ion transport and superoxide dismutase activity. However, since the second parental organism is still unidentified, it is unknown whether the observed expression divergence between homologs arose due to hybridization or whether it was already existing between orthologs of the parental species. One limitation of our study is that the conditions used do not fully resemble those of infection. However, previous studies have reported that the transcriptional profile of Candida albicans growing in yeast extract-peptone-dextrose (YPD) medium is remarkably similar to the profile during interaction with the host during infection (43). Thus, although it remains to be clarified whether the observed transcriptomic differences are actually related to differences in virulence, we argue that this possibility should be considered in future studies.

### Conclusions.

Altogether, our analyses provide evidence of a moderate effect of hybridization on the transcriptome of pathogenic hybrid yeasts, in line with previous observations for other fungal hybrids. Interestingly, the significant overlap of genes in heterozygous blocks, including DE and ASE genes, in the two independently formed hybrids suggests the existence of selecting constraints acting on genes that show altered expression in the hybrid. Similarly, we detected that an increase in LOH was associated with higher fractions of ASE genes, suggesting that ASE genes are preferentially retained in heterozygosis in the hybrid. Finally, we detected ASE genes in the pathogen studied which are known to have direct implications in fungal virulence in other yeast species, like C. albicans, making them a target for further studies of the emergence of fungal pathogenicity.

## MATERIALS AND METHODS

### Strains.

We analyzed three diploid strains of C. orthopsilosis, strains MCO456, CP124, and 90-125, with the latter belonging to the lineage of one of the putative parental strains of the two former hybrid species (13, 36, 44).

### Experimental conditions and RNA extraction.

RNA extraction was performed on the samples growing at the exponential phase in rich yeast extract-peptone-dextrose (YPD) medium at 30°C.

The experiments were performed as follows. First, we measured growth curves for each individual strain to delimit the mid-exponential growth phase. For this, each strain was plated on a YPD agar plate and grown for 3 days at 30°C. Single colonies were cultivated in 15 ml YPD medium in an orbital shaker (30°C, 200 rpm, overnight). Then, each sample was diluted to an optical density at 600 nm (OD_600_) of 0.2 in 50 ml of YPD and grown for 3 h under the same conditions. Samples were diluted again to an OD_600_ of 0.1 in 50 ml of YPD to start all experiments with a similar amount of cells. Cultures were grown at 30°C for 24 h,  and OD_600_ was determined every 60 min with a TECAN Infinite M200 microplate reader. Once the cells reached the mid-exponential phase, the protocol was repeated until all samples were growing at the exponential phase. Then cultures were centrifuged at 16,000 × *g* to harvest 3 × 10^8^ cells per sample. Total RNA from all samples was extracted using the RiboPure RNA yeast purification kit (ThermoFisher Scientific) according to the manufacturer’s instructions. Total RNA integrity and quantity of the samples were assessed using the Agilent 2100 Bioanalyzer with the RNA 6000 Nano LabChip kit (Agilent) and NanoDrop 1000 Spectrophotometer (ThermoFisher Scientific).

### RNA-Seq library preparation and sequencing.

Sequencing libraries were prepared using the TruSeq Stranded mRNA Sample Prep kit v2 (RS-122-2101/2; Illumina) according to the manufacturer’s instructions (unless specified otherwise). One microgram of total RNA was used for poly(A) mRNA selection using streptavidin-coated magnetic beads. The samples were then fragmented to approximately 300 bp, and cDNA was synthesized using reverse transcriptase (SuperScript II; Invitrogen) and random primers. The second strand of the cDNA incorporated dUTP in place of dTTP. Double-stranded DNA (dsDNA) was further used for library preparation. dsDNA was subjected to A-tailing and ligation of the barcoded Truseq adapters. All purification steps were performed using AMPure XP beads (Agencourt). Library amplification was performed by PCR on the size-selected fragments using the primer cocktail supplied in the kit. To estimate the quantity and check the fragment size, libraries were analyzed using Agilent DNA 1000 chip (Agilent), and were subsequently quantified by quantitative PCR (qPCR) using the KAPA Library Quantification kit (Kapa Biosystems) prior to amplification with Illumina’s cBot. Libraries were loaded and sequenced using 2 × 50 or 2 × 75 read lengths on Illumina’s HiSeq 2500. Experiments were performed in three biological replicates. All library preparation and sequencing steps were performed at the Genomics Unit of the Centre for Genomic Regulation (CRG), Barcelona, Spain.

### Bioinformatics analysis. (i) Quality control of sequencing data.

We used FastQC v0.11.6 (http://www.bioinformatics.babraham.ac.uk/projects/fastqc) and Multiqc v. 1.0 (46) to perform quality control of raw sequencing data.

### (ii) Phasing of heterozygous genomic regions.

The reference genome and genome annotations for the putative parent strain C. orthopsilosis 90-125 were obtained from NCBI (assembly ASM31587v1, last accessed on 8 December 2018). To assess allele-specific expression in the hybrid strains, we phased the hybrid genomes following the procedure illustrated in Fig. 2 and described further below.

Specifically, we first phased (i.e., resolved alternative haplotypes) the genes located in the heterozygous regions in the MCO456 and CP124 hybrid strains. To do this, we used DNA sequencing data of these strains (SRA accession numbers ERR321926 [12, 13] and SRR3547561 [11]). We first trimmed these data using Trimmomatic v. 0.36 software with default parameters and subsequently processed the data using mapping and variant calling modules of HaploTypo v.1 pipeline, setting the filter of single nucleotide polymorphism (SNP) clusters at 5 SNPs in a 20-bp window (47), and subsequently removed indels using vcftools v0.1.16 (48). Then, using the heterozygous variants, we defined heterozygous blocks using bedtools v2.29 (49) with the *merge* function as described in reference 12 and further optimized in reference 10—if the distance between two heterozygous variants is less than 100 bp, that region constitutes a heterozygous block, if the next variant to that block is located in less than 100 bp, the block is extended, otherwise it is interrupted. After defining heterozygous blocks, we identified genes located within the blocks using a custom python script find_genes_in_heterozygous_blocks.py v1.0 (available at https://github.com/Gabaldonlab/C.-orthopsilosis-ASE/blob/master/find_genes_in_heterozygous_blocks.py). Samtools v. 1.9 was used to index the reference genome. Sorting of vcf files was done by *sort* function in bash. Subsequently, we inserted the alternative variants of the genes located in heterozygous blocks in the reference genome of the 90-125 strain using GATK v.3.7 (50), thus reconstructing the sequence of the alternative parental genome within the defined heterozygous blocks.

### (iii) RNA-Seq and allele-specific expression analysis.

Transcriptome sequencing (RNA-Seq) read mapping and summarization were performed using the splice junction aware mapper STAR v. 2.7.3a (51). GFF-to-GTF format conversion for genome annotations was done using the gffread v. 0.11.6 (52) utility. For strain 90-125, we mapped RNA-Seq data to the 90-125 reference assembly, while for the hybrid strains, the data were mapped to a concatenated reference genomes, obtained by combining the 90-125 reference and the reconstructed parental reference. For mapping to the concatenated reference, we set the STAR option --outFilterMismatchNmax to 0 to restrict the mismatches in read alignments.

Differential gene expression and allele-specific expression were assessed using DESeq2 v. 1.22.2 (53). For allele-specific expression comparisons, the sizeFactors were set at 1 for all the samples, since the read counts for alleles come from the same library. For a gene to be considered differentially (allele-specifically) expressed, we used a threshold of |log_2_ fold change (L2FC)| > 1.5 and padj (adjusted *P* value) of < 0.01. Hypergeometric tests were performed using the phyper function of R with lower.tail and log.p parameters set at FALSE.

To visualize gene expression data, we used ggplot2 v. 2_3.0.0 R library (54). Putative functions of C. orthopsilosis genes were retrieved from the *Candida* Genome Database (55).

### Data availability.

RNA-Seq data are deposited at the SRA database under accession numbers SRR10251160 to SRR10251168.
